# The importance of methionine metabolism

**DOI:** 10.7554/eLife.47221

**Published:** 2019-05-02

**Authors:** Ramon I Klein Geltink, Erika L Pearce

**Affiliations:** 1 Department of ImmunometabolismMax Planck Institute of Immunobiology and EpigeneticsFreiburgGermany

**Keywords:** lymphocyte, T cell activation, nutrient uptake, methionine metabolism, Mouse

## Abstract

T helper cells import the amino acid methionine to synthesize new proteins and to provide the methyl groups needed for the methylation of RNA and DNA that drives T cell proliferation and differentiation.

**Related research article** Sinclair LV, Howden AJM, Brenes A, Spinelli L, Hukelmann JL, Macintyre AN, Liu X, Thomson S, Taylor PM, Rathmell JC, Locasale JW, Lamond AI, Cantrell DA. 2019. Antigen receptor control of methionine metabolism in T cells. *eLife* **8**:e44210. doi: 10.7554/eLife.44210

The immune system relies on a number of different cells types that work together to detect and clear unwanted infections from the body. T helper cells (also known as CD4^+^ T cells, and hereafter referred to as T cells) play an important role in this process as they modulate the activity of the immune cells that rid the body of infections. Antigens on the surface of infected cells can activate different subpopulations of T cells by binding to antigen-specific receptors on the surface of the T cells (Figure 1). Once activated, the T cells proliferate rapidly and work together to mediate the immune response against the antigen.

**Figure 1.**
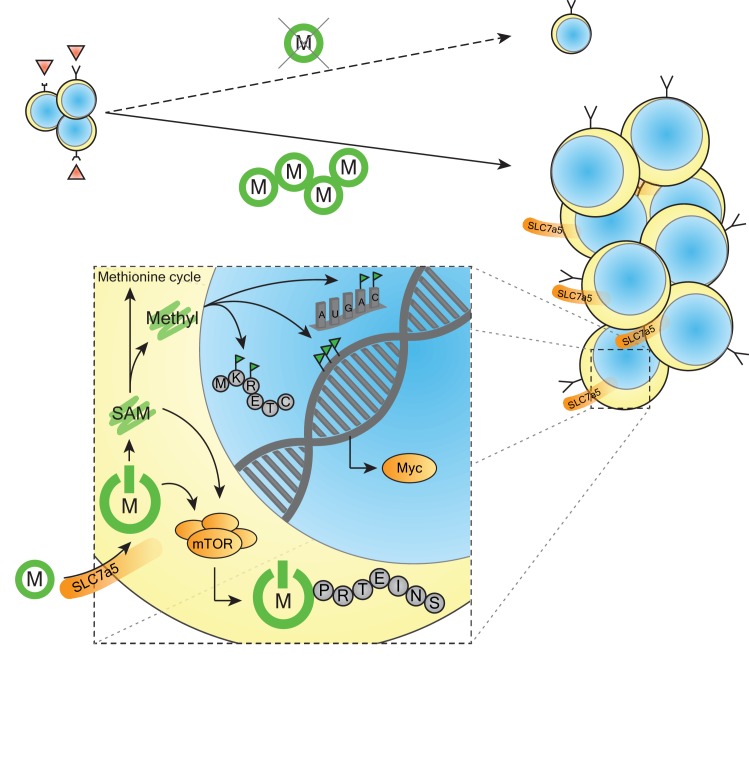
Methionine and T cells. When T cells recognize an antigen (red triangles; top left) they become activated and expand in both number and size. This expansion is dependent on the essential amino acid methionine (green circled M). In the absence of extracellular methionine, the T cells fail to expand (dashed line and arrow). When extracellular methionine is present, it is imported into the activated T cell by the system-L amino acid transporter Slc7a5. Inset: once inside the cell, the methionine (green start symbol M) is able to start the synthesis of new proteins through activation of the protein kinase mTOR. In addition, enzymatic conversion of methionine to s-adenosylmethionine (SAM) can also activate mTOR, and is used to donate methyl groups for the methylation of RNA residues (A: adenosine; C: cytosine), histones, DNA, and specific amino acids in proteins (K: lysine; R: arginine). The enhanced methionine import and generation of methyl groups can enhance the expression of the transcription factor Myc. Green color indicates methionine or methionine-derived metabolites; orange depicts activation or upregulation.

This rapid expansion of T cells is, however, metabolically taxing because DNA, proteins and other biomolecules have to be produced prior to every division of the cells. The synthesis of new proteins during this period relies on the T cells importing the essential amino acid methionine. In addition to its role in protein synthesis, methionine can also enter the 'methionine cycle' and be converted into s-adenosylmethionine (SAM), which provides methyl groups for numerous biochemical reactions.

SAM is a substrate for methyltransferase enzymes that are involved in the methylation of many different molecules (see Figure 1). The addition of a methyl group to a histone protein, for example, can alter gene transcription (Allis and Jenuwein, 2016); the methylation of other proteins influences processes such as signal transduction and metabolism (Murn and Shi, 2017); and the methylation of RNA can have a significant influence on gene expression (Zhao et al., 2017). Now, in eLife, Linda Sinclair and Doreen Cantrell, both at the University of Dundee, and co-workers at Dundee, Vanderbilt University and Duke University report that in addition to importing methionine for protein synthesis, activated T cells use it to generate the methyl groups needed for the methylation of DNA and RNA – processes that drive the differentiation and proliferation of T cells (Sinclair et al., 2019).

Using high-resolution mass spectrometry and metabolic labeling, Sinclair et al. showed that the activation of T cells by antigens led to a rapid upregulation of a methionine transporter called Slc7a5. The antigen activation of T cells also led to a marked increase in methyltransferases: however, the ability of these enzymes to methylate DNA, RNA or a protein depends on the availability of SAM. Since the expression of the enzymes that control the level of SAM do not change as a result of T cell activation, Sinclair et al. conclude that the import of methionine through Slc7a5 is the rate-limiting factor for the generation of methyl groups during T cell activation.

The expression of receptors for IL2 – a growth factor that drives proliferation of T cells – is also increased in response to antigen engagement with T cell receptors. Sinclair et al. observed that IL2 receptors were still upregulated even in the absence of methionine or the methionine transporter. This suggests that activation signals prepare cells to utilize extracellular methionine, but that the rapid upregulation of Slc7a5 and import of methionine is needed for the full activation of T cells.

Previous studies have shown that two other proteins – the protein kinase mTOR, which is involved ribosome biogenesis, protein translation and a number of other processes (Sabatini, 2017); and the transcription factor Myc – have important roles in regulating metabolism in T cells (Powell et al., 2012; MacIver et al., 2013), as does the upregulation of the nutrient transporters that import key metabolic building blocks such as glutamine and leucine. The fact that the activation of mTOR also enhances the expression of Myc and an amino acid transporter called CD98 illustrates the close connections between metabolic reprogramming and amino acid availability in immune cells (Wang et al., 2011; Sinclair et al., 2013). Sinclair et al. also showed that mTOR is partially dependent on the import of methionine to drive protein synthesis and fully activate the T cells.

It was also know that Slc7a5 imports leucine (in addition to importing methionine), and that the absence of either of these amino acids in the T cell leads to suboptimal activation of mTOR. It is possible that the need for multiple metabolites to support differentiation and proliferation may be a way of preventing T cells being activated when they should not be. Given recent advances in single-cell analysis it is conceivable that researchers might one day be able to measure changes in the epigenome (and also the transcriptome, proteome, acetylome and methylome) of T cells and combine these results with measurements of the flux through metabolic pathways to better understand the dynamic changes in T cells that underlie immune function. While aspects of the study by Sinclair et al. highlight the complexity of the metabolic reprogramming that T cells undergo during activation and differentiation, it also moves the field forward by establishing how the essential amino acid methionine supports T cell function. Precisely how levels of dietary methionine will contribute to methionine uptake by T cells – and whether those levels are modulated during infection, cancer or autoimmunity to influence T cell responses – is not yet known and will require further study.
